# Implicit Processing of Numerical Order: Evidence from a Continuous Interocular Flash Suppression Study

**DOI:** 10.3390/jintelligence11050096

**Published:** 2023-05-16

**Authors:** Dana Sury, Orly Rubinsten

**Affiliations:** 1 Department of Learning Disabilities, Faculty of Education, Beit Berl College, Kfar Saba 4490500, Israel; 2 Edmond J. Safra Brain Research Center for the Study of Learning Disabilities, Department of Learning Disabilities, University of Haifa, Haifa 3498838, Israel

**Keywords:** order processing, numerical cognition, enumeration, continuous flash suppression

## Abstract

Processing the ordered relationships between sequential items is a key element in many cognitive abilities that are important for survival. Specifically, order may play a crucial role in numerical processing. Here, we assessed the existence of a cognitive system designed to implicitly evaluate numerical order, by combining continuous flash suppression with a priming method in a numerical enumeration task. In two experiments and diverse statistical analysis, targets that required numerical enumeration were preceded by an invisibly ordered or non-ordered numerical prime sequence. The results of both experiments showed that enumeration for targets that appeared after an ordered prime was significantly faster, while the ratio of the prime sequences produced no significant effect. The findings suggest that numerical order is processed implicitly and affects a basic cognitive ability: enumeration of quantities.

## 1. Introduction

Humans and even animals use numbers on a daily basis. Simple activities such as buying groceries or estimating the number of people in a room involve daily numerical knowledge. Numerical concepts involve different attributes, including cardinality and ordinality. Accordingly, numerical knowledge requires representations of both quantities and ordinal relationships (Jacob and Nieder 2008; Nieder 2005). While processing of cardinality is defined as a core cognitive system (e.g., Carey 2009; Feigenson et al. 2004; Spelke and Kinzler 2007), order processing has received much less attention in contemporary scientific and clinical approaches. Here, we investigate an innovative outlook that argues for the existence of a cognitive system designed to evaluate numerical order. Note that we define numerical order as a state, or more specifically, a reasonable pattern between individual or continuous elements of items of a group. This definition is based on findings on the processing of numerical and non-numerical ordinality suggesting ordinality may refer not only to the positions within the sequence but also to the relationships between elements (for a review, see Sury and Rubinstan 2011). The traditional definition of the numerical ordinal aspect commonly refers to a single item in a sequence and defines ordinality as the relative position or rank of a number (e.g., Vogel et al. 2017, 2019). Our definition expands this understanding by claiming ordinality is a sequential feature (e.g., a decrease or increase in a quantity by the same ratio or distance) rather than a single item. Hence, our definition refers to the regularities or, in other words, the constancy of the relationships between magnitudes in the sequence. From recognizing speech to generating muscle movement, relationships must be processed within series of items (de Hevia 2021; Dehaene et al. 2015; Namboodiri et al. 2015). However, little is known about how the human mind encodes ordered relationships and the effect of this encoding on other basic cognitive processes. Moreover, the ability to process order is not currently recognized as a system that is implicitly employed (i.e., implicit processes are basic functions that involve automatic activation of information and are carried out inattentively (De Houwer and Moors 2012; Kouider and Dehaene 2007; Reber 1989; Schneider and Shiffrin 1977)).

In the numerical domain, the order of numbers is a salient parameter specifically in the symbolic sequence (Dehaene et al. 2015; Lyons and Ansari 2015). However, current opinion is that numerosity and not order is the basis of the numerical core system. Nonetheless, several studies have demonstrated order processing in non-human primates (Cantlon and Brannon 2006), human infants (Brannon 2002; de Hevia 2021; de Hevia et al. 2017; de Hevia and Spelke 2010; Fló 2021; vanMarle 2013), and even animals that are phylogenetically distant from humans (e.g., rats (Suzuki and Kobayashi 2000), parrots (Pepperberg 2006), fish (Petrazzini et al. 2015), bees (Bar-Shai et al. 2011), and jackdaws (Pfuhl and Biegler 2012)). These studies provide clues to an evolutionarily based order input analyzer. The aim of the current study was to investigate the human ability to implicitly, inattentively, and immediately perceive order (as opposed to active, attentive, and exact computation) between non-symbolic quantities.

### 1.1. Implicit Processing of Ordinality

Processing order or the arrangement of items in a particular sequence may be perceived as a conscious cognitive process that requires rule-following computations or, in other words, explicit processing (i.e., requiring intentional and conscious cognitive activity). However, evidence for the existence of an evolutionarily based order input analyzer naturally leads to the question: Do humans implicitly process order? The answer to this question remains unclear mainly because current findings on the pre-verbal ability to process order are studied and interpreted in terms of active (i.e., require attentional resources) explicit processing of order relations (e.g., Bregman and Campbell 1971; Cantlon and Brannon 2006; de Hevia et al. 2017). In the current study, using a masked priming technique known as “Continuous Flash Suppression masked priming,” that allows subliminal presentations that last seconds (Yang and Blake 2012), we aimed to explore the possibility of implicit order processing and its relationship to numerical skills, specifically numerical enumeration.

### 1.2. Ordinality and Numerosity

It has been argued that the order of number symbols is essential for symbolic number processing and even serves as a stepping stone from approximate magnitude representation to mathematical competence (Lyons and Beilock 2011; Sommerauer et al. 2020). Hence, number symbols clearly represent not only numerical sequences (i.e., order) but also numerical magnitude (i.e., cardinality; the total number of items in a set) or, as will be termed here, numerosity.

A widely accepted view is that numerosity stands at the base of the fundamental sense of quantity. Processing numerosity is considered a core system (Nieder and Dehaene 2009; Piazza 2010) that enables humans and other animals to implicitly estimate non-symbolic numerical information and represent the approximate numerical value of a collection of objects (Cantlon et al. 2009; Viswanathan and Nieder 2013). For example, Ferrigno et al. (2017) and Cicchini et al. (2016) demonstrated that humans universally and spontaneously extract numerical information. However, it seems that human nonverbal numerical perception is enhanced by symbolic numeracy (Ferrigno et al. 2017; Sella and Lucangeli 2020). A major signature of numerosity is the ratio effect: in comparison tasks (most commonly used to measure numerosity), accuracy falls and reaction time (RT) increases as the ratio of the numbers to be compared approaches one (Dehaene 1997). The ratio effect appears even when it is irrelevant for the task at hand; thus, numerical estimation is considered to be automatically or implicitly activated (Cohen Kadosh et al. 2008; Henik and Tzelgov 1982; Rubinsten and Henik 2005). Even after different kinds of methodological manipulations, a numerical ratio effect is present in non-symbolic numerical tasks; thus, the numerical ratio effect is considered strong evidence that individuals are basing their non-symbolic magnitude estimation on *numerical* information (Halberda et al. 2008; Leibovich et al. 2013; Pinhas et al. 2023; Reynvoet et al. 2002). Nonetheless, it should be noted that comparisons or priming tasks that address how a participant processes relationships between *two* numerals (Dehaene et al. 1998; Koechlin et al. 1999; Roggeman et al. 2007) cannot actually dissociate order and numerical processing. To carry this out, and to clearly dissociate order and numerical processing, we manipulated numerical ratio to learn about numerical processing and simultaneously presented three groups of dots to learn about order processing. Indeed, a complete dissociation of order and numerosity seems impossible because they are interwoven into the numerical sequence. That is, to compare two quantities or to know a single quantity requires having knowledge about how this quantity evolves, i.e., knowledge about the order within and between quantities (Dehaene et al. 2015). Hence, the ratio effect might in fact result from the ability to process ordinal relationships (i.e., order) and not only quantities (i.e., numerosity). For example, in Cicchini et al. (2014), adult participants were asked to indicate the quantity of an array of dots on a line demarcated by two sample numerosities. The results indicated a strong relationship between number line mapping in the current trial and the magnitude of the previous trial. Hence, the judgment of a single quantity is dependent on the order in which quantities are presented. Similarly, Corbett et al. (2011) showed that the perceived number of an array of objects depends on the perceived number of objects in previous displays.

Using event-related potentials combined with an order judging task (i.e., judging the order of a sequence of three non-symbolic quantities such as groups of dots), Rubinsten et al. (2013) dissociated order from numerosity. Their results showed that while the numerical ratio was associated with a later scalp medial posterior positivity (130–200 ms), order estimation was associated with early scalp parietal and lateral occipital positivity (80–130 ms). Taken together, an accumulating body of evidence suggests that order may play a crucial role in numerical cognition and serve as a stepping stone to the human ability to estimate quantity. However, few studies have examined non-symbolic numerical order processing (Lyons and Beilock 2013; Rubinsten et al. 2013; Rubinsten and Sury 2011; Sury and Rubinsten 2019), even though the processing of non-symbolic numerals is considered a core pre-verbal ability and possibly the underlying framework of subsequent mathematical ability (Nieder and Dehaene 2009). If an implicit-based order input analyzer indeed plays a crucial role in numerical cognition (Cantlon et al. 2009; Piazza 2010), then non-symbolic numerical order should be implicitly processed.

### 1.3. The Current Study

The present study challenges the view that the core representation of quantities relies on a non-symbolic numerical system that represents the approximate numerical value alone. Specifically, it focuses on three main questions: (1) Is there a cognitive system designed to evaluate order (i.e., sequential regularities between quantities), and if so, (2) does this system involve implicit processing, similar to numerosity? Finally, (3) does this system affect the processing of numerosity?

Based on evidence suggesting that order processing appears early in human development and shares a cerebral basis with numerosity (de Hevia 2021; Fló 2021; Gevers et al. 2003; Ischebeck et al. 2008; Kaufmann et al. 2009; Suanda et al. 2008), we predicted that order could be estimated implicitly.

With regard to the three major research aims, we employed a masking technique using interocular continuous flash suppression (CFS) (Tsuchiya and Koch 2005). CFS is a psychophysical method for studying visual perception that allows subliminal presentations that may last up to a minute (Sklar et al. 2012; Stein et al. 2020; Yang and Blake 2012). The technique’s name refers to a binocular rivalry display: a visual stimulus is presented to one eye while a mask (i.e., a rapidly changing sequence of high-contrast, contour-rich patterns) is presented to the other eye. The rapidly changing mask dominates awareness and allows suppression of the target stimulus (Tsuchiya and Koch 2005).

With implicit measures such as priming combined with CFS, mental content can be assessed without requiring awareness of the relationship between the response and the measured content (i.e., order). In the current study, in two experiments, adult participants were presented with a prime sequence (i.e., three groups of dots presented simultaneously, which acted as the investigated stimulus) to one of their eyes. This sequence competed with a dynamic (10 Hz) mask pattern presented to the other eye. The invisible prime sequences could be ordered or not-ordered. After the prime and mask disappeared, the target stimulus appeared and was presented to both eyes. The target included a single quantity. Participants were asked to enumerate how many dots were presented in the single circle acting as the *target* stimulus (numerosity). We used implicit (i.e., objective test block; experiment 1 and 2) and explicit (individually adjusting prime contrast) measures to ensure lack of participant awareness of the prime. If order is indeed implicitly processed, then ordered primes may affect the estimation of the target quantity that is actually the fourth item of the sequence.

Two variables (i.e., characteristics of the sequence) were systematically manipulated: (1) order [ordered, including both ascending (left to right) and descending (right to left) sequences, vs. non-ordered quantities as primes] to study implicit processing of order and its effect on numerical enumeration; and (2) ratio (big or small ratios between each two adjacent groups of dots) to study the processing of core numerical knowledge and its relationship with ordinality. Our main hypothesis was that not only would the typical ratio effect be found (Cohen Kadosh et al. 2007; Rubinsten and Henik 2005; Tzelgov et al. 1992) but a main effect of order would also be identified. That is, we hypothesized that ordered sequences would induce a faster response to the subsequent target and predicted that this order effect would appear independently of the ratio and thus suggest implicit estimation of order. In experiment 1, we employed the CFS masked priming task using strict analysis (i.e., a multilevel model, e.g., Heck and Thomas 2015; Heck and Reid 2017) comparing performance patterns of the ‘unaware’ with those of the ‘aware’ group. In experiment 2, we aimed at replicating findings from experiment 1. Moreover, following a criticism of the post hoc method for excluding participants used in experiment 1 (Shanks 2016), in the second experiment we employed a rigid method for ensuring invisibility of the prime (i.e., contrasts of the prime sequences were adjusted individually for each participant following a staircase procedure). This individual adjustment of the prime contrast in experiment 2 was performed to ensure maximal stimulus strength even under full suppression. Further, in order to strengthen our hypothesis, we also calculated Bayes factors, which quantify the likelihood of one hypothesis (H0) over the other (H1) for each analysis (e.g., Wagenmakers et al. 2017). Thus, current findings relied on both statistical frequencies and probability evidence.

## 2. Experiment 1

### 2.1. Material and Method

#### 2.1.1. Participants

A total of 72 healthy adult students were recruited from Haifa University (mean age = 27.5 ± 4.1, 50 females and 22 males), all with intact vision (i.e., did not require or wear glasses or contact lenses). Participants gave written consent to participate in the experiment and received monetary compensation for their participation, and the overall procedure was approved by the ethics committee of the University of Haifa. All methods were performed in accordance with the relevant guidelines and regulations of this committee.

To ensure unawareness of the prime, a forced-choice discrimination test was introduced to participants (Bargh and Chartrand 2000). As a result, 28 participants were excluded from further analysis because they showed significant above-chance performance for invisible stimuli in the control experiment or reported subjective awareness of the prime (see objective test block). Additionally, three participants were excluded from the analysis because of technical errors that caused failure to measure the RT of their vocal response.

Eventually, the data for 41 participants who did not show any awareness of the prime were analyzed and are reported in this paper (mean age = 26.3 ± 6.1, 30 females and 11 males).

#### 2.1.2. Experimental Task

Using the CFS technique, in each trial, participants were primed by three concurrently presented non-symbolic quantities (i.e., three groups of dots presented simultaneously) to one of the participant’s eyes, which competed with a dynamic (10 Hz) mask pattern presented to the other eye. The prime could be either ordered (ascending or descending direction, e.g., 3, 4, 6 or 6, 4, 3, respectively), not-ordered (i.e., no ordered relationship between the three items, e.g., 3, 6, 4), or neutral (the same quantity appeared three times, acting as “fillers”). After the prime and mask disappeared, the target stimulus appeared and was presented to both eyes. The target included a single circle with dots inside. Participants were required to enumerate how many dots were presented in the single circle acting as the target stimulus that appeared after the prime. The quantity of the target stimulus corresponded to the sequence presented in the prime (i.e., the fourth item in the sequence, e.g., nine dots appeared after the sequence of 3, 4, and 6 quantities).

#### 2.1.3. Stimuli

##### Prime Stimuli

Prime stimuli (presented to one eye) consisted of multiple-dot patterns ranging from 1 to 20 dots per stimulus (Table 1). Dots were white and appeared on a black background presented within a white visible circle of 4° visual. To ensure that participants related only to quantities in the current task, low-level visual features (i.e., density and area of the dots) were randomized. The three groups of dots in each stimulus were presented along a (non-visible) horizontal axis, with the central pattern located in the center of the screen. For a detailed description of the numerical stimuli and the randomization of visual feature conditions, see Appendix A.

The quantities of the three groups of dots were ordered in an ascending direction (i.e., small, medium, large) or descending direction (i.e., large, medium, small) in a non-ordered, random sequence [that included two possible presentations: (1) medium, small, large quantities (e.g., 4, 2, 6) or (2) small, large, medium quantities (e.g., 2, 6, 4)] or a neutral sequence (neutral sequences act as “fillers”). In the neutral sequences, for each numeral that appeared in the prime sequences, a neutral prime appeared that was composed of three circles surrounding three identical quantities. While quantities were identical, stimulus were different (i.e., different dot pattern) and each stimulus did not appear more than once in the experiment. In each stimulus, the ratio between every two adjacent groups of dots was manipulated in order to assess the implicit processing of numerosity. Participants were presented with a ratio of 0.7 or 0.5 between quantities presented in the stimulus (Table 1).

##### Mask Stimuli

Mask stimuli (presented to the other eye) were patterns of randomly assigned colored circles, consuming half the screen and changing randomly at a rate of 10 Hz.

##### Target Stimuli

Target stimuli consisted of multiple-dot patterns that represented the fourth item of the sequences presented as prime (e.g., a quantity of nine dots to complete the 3, 4, 6 dot sequence; Table 1). The dots were white and appeared on a black background presented within a white visible circle of 4° visual. Target stimuli appeared binocularly, in the center of both sides of the screen.

#### 2.1.4. Apparatus

Stimuli were presented on a cathode ray tube (CRT) monitor using E-Prime 2.0 software. The CRT monitor was fitted with a mirror stereoscope to allow stimuli to be presented monocularly.

#### 2.1.5. Procedure

##### CFS Ordered Priming Task

**Figure 1:**
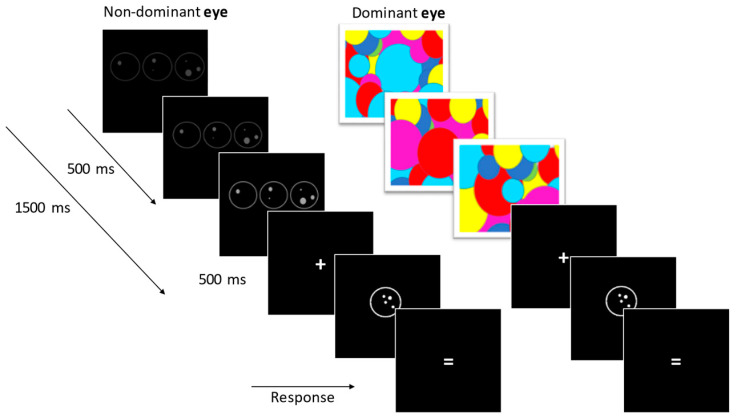
Schematic representation of the experimental paradigm. The prime sequence was presented to one eye and competed with a dynamic (10 Hz) mask pattern presented to the other eye. The sequence was gradually ramped up in contrast during the first 500 ms of presentation.

Participants were told that they were performing an “enumeration task.” Each experimental trial began with the monocular presentation of a prime sequence [which was gradually ramped up in contrast (from 0% to 50%) during the first 500 ms of presentation] to one eye—the non-dominant eye—and a rapidly changing mask to the other eye. These were presented for 1500 ms. All primes were followed by the binocular presentation of a fixation (500 ms). Finally, a target stimulus (i.e., dot pattern) appeared binocularly, presented for 200 ms. After presentation of the target stimulus, the sign “=” appeared on the screen (indicating to the participants that they should report their estimation) until the microphone registered a voice response or for 5000 ms max. (Figure 1).

Participants were not informed of the presence of the prime. They were simply instructed to enumerate the quantity presented after a “signal” as quickly and accurately as possible. Five experimental blocks were presented (five repetitions for each trial) with a total of 320 trials. Each block contained 64 randomly assigned trials: 4 orders (ascending, descending, and 2 non-ordered types) × 2 ratios (0.7 and 0.5 ratio between items of the sequence in the prime stimuli) × 4 different prime sequences (Table 1) + 32 neutral sequences. The dependent measures were RT (reaction time) and participant enumeration.

##### Objective Test Block

To ensure unawareness of the primes, immediately after completion of the experimental blocks, a forced-choice objective test was administered. The objective block consisted of 128 trials (64 trial pairs), in which the presentation parameters were identical to the experimental trials. However, in this case participants were informed about the existence of primes. Although one half of the sequences were identical to those used in the experiment, while the other half did not contain a prime. A total of 128 trials were divided into 64 pairs in which one of the trials included a prime, while the other did not. The task was to indicate whether a prime appeared on the first or second trial in each pair. Upon completion of the objective test, participants were debriefed and asked directly whether they had seen the primes during the experimental blocks.

Binomial distribution was used to determine whether each participant performed better than chance on the objective block. Twenty-eight participants performed better than chance in the objective block (mean score for excluded participant = 65% accuracy rate, SD = 1.3%, mean score for included participant = 45% accuracy rate, SD = 0.8%). To note, successful suppression is strongly affected by individual differences (Sklar et al. 2012). Hence, even though the number of excluded participants may seem high, it falls within the normal range for long-duration CFS priming, in which successful suppression is strongly affected by individual differences (Greenwald et al. 1995; Sklar et al. 2012).

#### 2.1.6. Data Analysis

The mean enumeration by each participant for each target in each condition was calculated (e.g., the mean enumeration for target value 9 that appeared after ordered, ascending sequences with a ratio of 0.5). Responses in which enumerations were above or below 2.5 standard deviations from this mean were excluded from the analysis. Accordingly, 896 trials (6.8% of the total trials) were excluded (Osborne and Overbay 2004). To further minimize the effect of outliers, we excluded trials with anticipatory responses (RT < 100 ms) from the analysis. Accordingly, 11 trials (0.45% of total trials) were excluded (Whelan 2008). The dependent variable for the current analysis was RT. See Table 2 for description of accuracy and RTs for each target.

##### Modeling Strategy

Shanks (2016) argued that the post hoc method for excluding participants (used in the current study) might result in a regression to the mean effects. Accordingly, we addressed this concern in our analyses, following Shanks’s recommendation: “Rather than excluding data, analytic techniques can be employed that retain all data and adopt different logic to estimate unconscious processing” (Shanks 2016, p. 766).

More precisely, to ensure this was not the case in our findings and to estimate the effect of ordered primes on the response time, we developed a multilevel modeling strategy integrating all participants’ measurements of response time beyond their mean response time (e.g., both means and variability across measurements within each participant). Our approach allowed us to use a preliminary cutoff point for awareness (see objective test block) yet retain the full variability across measurements within each participant. To control for possible regression to the mean effect, we ran the model examining RTs and accuracy of enumeration as dependent measures and using order, ratio, and direction of prime sequences as independent variables, first across all participants with a grouping variable representing students below and above the cutoff. The variability across students with respect to the total variance is captured by the intra-class correlation (ICC) coefficient. The higher the coefficient (ICC > 0.05), the higher the role of the participants in potentially explaining their response time. We further ran an unawareness subset of the model to determine the order effect. If the first showed no order effect, while the second did, this means that the regression to the mean effect is minor, as mixed awareness and unawareness in the participant sample should show no difference, while the unawareness subset should, and vice versa for the effect of ratio (for a detailed description, see Appendix B). This approach differs from the more traditional approach in which the within subject variance is ignored. That is, the advantage of multilevel analysis lies in its recognition of variation within subjects or extracting the most out of the data. The analysis was carried out using Mplus V.8.0 software, which allows multilevel analyses, and provides the option for alternative estimators, e.g., MLR (Maximum Likelihood Rescaled), or Bayes estimator, among several alternatives.

Our multilevel approach included a regression analysis at two levels. At level 1, the dependent variables were the residual variance of RTs and accuracy; at level 2, the dependent variables were mean RT for each participant and the residual variance of RTs. The independent variables were the order of the sequences (i.e., ordered or non-ordered) and the ratio between items of the sequences presented (i.e., 0.7, 0.5).

### 2.2. Model Results and Discussion

**Figure 2:**
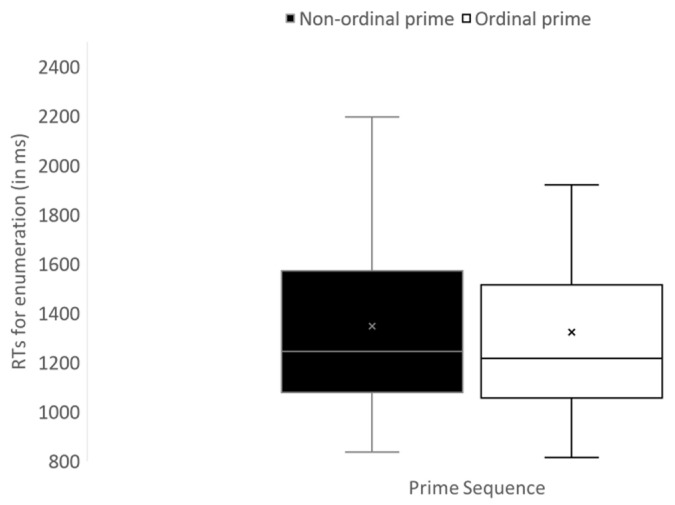
Experiment 1—boxplot for Mean RTs for enumeration of a target as a function of order of the prime sequences (error bars reflect minimum and maximum range).

*Preliminary ICC test*: we found that ICC for the unconditional response time was high for all students and for the unawareness subgroup, respectively (*n* = 69, ICC = 0.31; n = 41, ICC = 0.34). This indicates that clusters of subjects’ measurements cannot be ignored. Thus, the results of the ICC analyses justify the following multilevel analysis. Our regression models included the order and ratio effects at level one, and the accuracy (accuracy rates in the objective test block) at level two. Table 3 presents the model results. When order is tested across all participants, it shows no significant effect on response time, that is, response time does not differ across ordered versus unordered prime stimuli. The opposite case is present for ratio. When the effect of order is tested across the students defined as unaware, RTs of enumerations for targets that appeared after an ordered prime (1321 ± 373 ms) was significantly faster than enumerations for targets that appeared after a non-ordered prime (1346 ± 369 ms, (b = −0.023, *p* < .05 see Figure 2 for illustration)). The level two residual variance indicates that differences exist across participants in their performance, regardless of their prior accuracy score. However, the difference between RTs of enumerations for targets that appeared after an ordered 0.7 ratio sequence vs. an ordered 0.5 ratio sequence was not significant (b = −0.062). Altogether, these results lend support to the expected effect of order among the unaware students (order was implicitly estimated to affect the enumeration of the target). Results also reduce the possible bias of the regression to the mean effect, and show that a preliminary cutoff helps determine a clear order effect.

The result of experiment 1 clearly indicates that ordinality of the prime sequence was implicitly processed. In addition, the results also indicate that implicit processing of ordered primes significantly affects the very basic ability to enumerate a single quantity. Moreover, and contrary to our hypothesis, the ratio of the prime sequences did not significantly affect enumeration. Thus, implicit processing of numerosity was not supported by the current findings. These results are compatible with previous findings indicating no evidence for numerical priming under interocular suppression (Hesselmann and Knops 2014). However, in the very few studies that used CFS to study the non-symbolic ratio effect, results were inconclusive (e.g., Bahrami et al. 2010 vs. Hesselmann et al. 2015; Hesselmann and Knops 2014). Accordingly, to strengthen current findings and to directly tackle the inconclusiveness of previous findings (Bahrami et al. 2010; Hesselmann et al. 2015; Hesselmann and Knops 2014), in experiment 2, we aimed to both replicate the findings of experiment 1, which indicate implicit processing of order vs. no such processing of numerical ratio. Further, due to a recent critique of the post hoc method for excluding participants, used in experiment 1 (Shanks 2016), in experiment 2, individual adjustment of the contrast between the primes was used in order to ensure the invisibility of the prime under CFS.

## 3. Experiment 2

### 3.1. Materials and Method

The methods were the same as in experiment 1, except for the following differences:

#### 3.1.1. Participants

An a priori analysis to compute requires size was conducted using ‘Gpower’ (http://www.gpower.hhu.de/en.html). For paired sample *t*-test (two tailed) the power analysis revealed a minimum sample size of n = 26 with effect size of 0.5 (calculated on the basis of the difference between order and non-ordered prime found in experiment 1) with α = 0.05 and a power (1 − β) = 0.80. Thirty-one healthy adult students were recruited from Haifa University, all with normal or corrected-to-normal vision. Participants gave written consent to participate in the experiment and received monetary compensation for their participation. The experiment and the overall procedure were approved by the ethics committee of the University of Haifa. All methods were performed in accordance with the relevant guidelines and regulations of this committee.

Five participants were excluded because they showed significant above-chance forced-choice discrimination performance for invisible stimuli in the control experiment (see experiment 1, objective test block). Ultimately, the data for 26 participants who did not show any awareness of the prime were analyzed and are reported in this paper (mean age = 27.6 ± 4.2, 16 females and 10 males).

#### 3.1.2. Prime Contrast

Following Shanks’ (2016) criticism of the post hoc method for excluding participants, used in experiment 1, in experiment 2, we employed a more rigid approach to ensure prime invisibility under CFS. Contrasts of the prime sequences were adjusted individually for each participant following a staircase procedure, as follows: after a stimulus presentation (i.e., mask and prime sequence of the main experiment), the participants had to press a key to indicate whether the stimulus had been visible or not. Based on this response, the stimulus contrast was decreased or increased in the next trial following a logarithmic scale (1-up-1-down staircase). Each participant completed two staircases of 20 trials. The stimulus contrast in the main experiment was set to the highest stimulus contrast that the participant always judged as invisible in the pretest. This individual adjustment of the prime contrast was performed to ensure maximal stimulus strength even under full suppression. The resulting luminance contrast was 32% ± 10% (from the original contrast of the stimuli).

#### 3.1.3. Objective Test Block

Binomial distribution was used to determine whether each participant performed better than chance on the objective block. Seventeen participants performed better than chance in the objective block (mean score for excluded participants = 57% accuracy rate, SD = 14%, mean score for included participants = 43% accuracy rate, SD = 2.7%). See objective test block in experiment 1 for detailed procedure.

#### 3.1.4. Data Analysis

All enumeration of targets was within a 2.5 deviation (i.e., target numerosity/enumeration) from target numerosity (mean deviation = 1.18 ± 0.3) Similar to previous CFS studies (Bahrami et al. 2010; Hesselmann et al. 2015; Hesselmann and Knops 2014; Sklar et al. 2012), the dependent variable in the current analysis was RT of enumeration. To further minimize the effect of outliers, trials with anticipatory responses (RT < 100 ms) were excluded from the analysis. Accordingly, 13 trials (0.48% of total trials) were excluded (Whelan 2008). Mean RTs for enumeration of target numbers were calculated for each participant (see Table 4). These mean RTs were subjected to three analyses: (1) ordinality (ordered vs. non-ordered) of the prime was used as a dependent variable in *t*-tests for dependent samples, with neutral primes not included because the current study focuses on the difference between ordered and non-ordered prime effects; and (2) ratio (0.5 vs. 0.7) of the ordered prime was used as an independent variable in a *t*-test for dependent samples. This analysis was carried out with data from the ordered sequences alone because the ratio of the non-ordered sequences was not preserved (i.e., the ratio 0.5 or 0.7 was not accurately preserved). When sequences are arranged in a “non-ordered” fashion, but still retain the exact number of items for each stimulus in the sequence, it is not possible to preserve a ratio between items that is similar to the ratio in the ordered sequences. (3) We further aimed to assess whether direction of the ordered primes is implicitly processed. Hence, direction (ascending vs. descending) of the ordered primes was used as a dependent variable in a *t*-test for dependent samples. This analysis as well was carried out only with data from the ordered sequences because non-ordered sequences do not hold a specific direction.

##### Bayesian Statistical Analyses

Additionally, to further test and quantify the strength of the statistical evidence of our hypotheses, Bayesian statistical analyses were performed and Bayes factors are reported for the critical tests (Wagenmakers et al. 2017). The Bayesian analyses were performed using JASP (JASP Team 2017).

A Bayes factor quantifies and indicates the likelihood of one hypothesis over another. As an aid for interpretation of the Bayes factors, we employed Jeffreys’s (1961) classification scheme: Bayes factors between 1/3 and 3 are labeled anecdotal evidence, Bayes factors between 3 and 10 (or between 1/3 and 1/10) indicate moderate evidence, and Bayes factors greater than 10 or smaller than 1/10 indicate strong evidence (Jeffreys 1961; Wagenmakers et al. 2017).

### 3.2. Results and Discussion

#### 3.2.1. The Significant Effect of Implicitly Presented Ordered Sequences on Numerical Enumeration (i.e., Main Effect of Order of the Primes)

**Figure 3:**
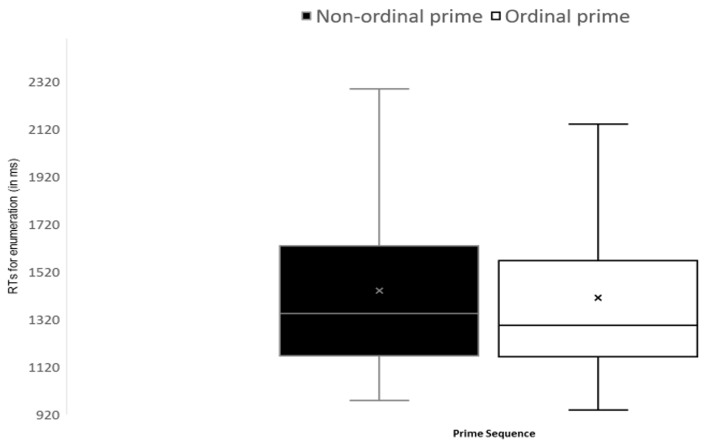
Experiment 2—a boxplot of Mean RTs for enumeration of a target as a function of the order of the prime sequences (error bars reflect minimum and maximum range).

The difference between RTs of enumerations of targets that appeared after a non-ordered prime (1439 ± 393 ms) vs. an ordered prime (1409 ± 391 ms) was significant [*t* (25) = 2.978, *p* = .006, *BF*_10_ = 13.79; Figure 3].

#### 3.2.2. The Non-Significant Effect of Implicitly Presented Numerical Ratios on Numerical Enumeration (i.e., the Main Effect of Ratio of the Ordered Primes)

The difference between RTs of enumerations for targets that appeared after an ordered 0.7 ratio sequence vs. an ordered 0.5 ratio sequence was non-significant [*t* (25) = 1.755, *p* = .079, *BF*_10_ = 0.904].

#### 3.2.3. The Non-Significant Effect of an Implicitly Presented Different Direction on Numerical Enumeration (i.e., the Main Effect of Direction of the Ordered Primes)

The difference between RTs of enumeration for targets that appeared after ordered ascending (left to right) sequences vs. ordered descending (right to left) sequences was non-significant [*t* (25) = −0.225, *p* = .824, *BF*_10_ = 0.212].

### 3.3. Discussion

The results of experiment 2 replicate the priming effect of order on enumeration of a single quantity. The absence of an effect of ratio or direction of the ordered prime on enumeration of a single quantity is also replicated. Thus, by employing a more rigid control on prime visibility and using Bayesian analysis, we have shown that numerical order is implicitly, estimated and in the current paradigm, the only characteristic of the prime sequences that affects enumeration of a single quantity.

## 4. General Discussion

In two experiments, using a masked priming technique, rigid method for ensuring prime invisibility under CFS and diverse statistical analysis, we show that order is clearly implicitly processed. Moreover, in both experiments, the results did not indicate an implicit processing of the ratio between each two adjacent items in the prime sequence of the ordered primes. These findings suggest that order acts as the salient numerical feature that is inattentively processed during estimation.

From the progression of the day (as sunlight increases and decreases gradually) to the Fibonacci sequences in nature, many objects and events in the world around us can be defined or characterized using order and sequential terms. This “natural role” may indeed affect how humans perceive the world. Combined with the current results, an intuitive notion is that during processing, humans may order the stimuli they perceive, or as Zacks and Taversky (2001) suggested, humans perceive events (and possibly other features) as having orderly relations despite the chaos and flux in the world. Such a tendency to order perceived information can explain the efficient processing of ordered vs. non-ordered numerical sequences found in previous studies (Lyons and Ansari 2015; Sury and Rubinstan 2011; Sury and Rubinsten 2019). One can argue that because ordered sequences are more compatible with cognitive expectations or processes, they are more efficiently processed. However, the current study suggests that human adults implicitly distinguish between ordered and non-ordered numerical sequences and hence are also tuned to non-ordered or chaotic relations in the human visual field. This ability to implicitly distinguish between ordered and non-ordered relations may affect how humans interpret information from the external world and execute a decision or an action based on it.

Indeed, order is commonly used to describe the perception of a sequence that is embedded in time (i.e., what comes before or after), such as a sequence of events (Hogan 1978), a mental time line (Bonato et al. 2012), sounds (Bregman and Campbell 1971), and language (Dehaene et al. 2015; Fló 2021); however, the current results suggest that order may affect the processing of simultaneously presented stimuli as well. Additional examples of this hypothesis are provided by face recognition (Gilad et al. 2009), three-dimensional space (Koenderink et al. 2002), depth (Nguyen et al. 2005), and numbers (Brannon and Terrace 2000). As early as the 1950s, in fact, Lashley (1951) argued that even animals grasp and use their knowledge on abstract multi-item sequential structures. Additionally, not only humans but also non-human animals demonstrate an ability to process the order that exists in a simultaneously presented sequence (Brannon 2002; Dere et al. 2005; Terrace 2005). Such findings demonstrate the role of order processing in various cognitive abilities.

To “connect these dots,” we propose the innovative perspective whereby the development of brain systems critically involved in the perception of action, space, and numerical cognition (Walsh 2003) depends on an initial ability to evaluate order. This cognitive system is tuned to efficiently pick up order information by *implicitly* capturing sequential regularities in the surrounding world. Importantly, we put forth the claim that human numerical intelligence significantly relies on this order input analyzer.

Below, we discuss the innovative conceptualization of the existence of a cognitive system designed to evaluate order. We show that the current findings also support the idea that this system may stand at the heart of numerical cognition as well as of other cognitive functions.

### Numerical Order Is Implicitly Processed

Our findings show that participants implicitly dissociate ordered from non-ordered numerical sequences. This result is reflected in the different response times to enumerations conducted after implicitly presented ordered vs. non-ordered sequences. Specifically, RTs were significantly faster when targets followed an implicit presentation of an ordered vs. non-ordered sequence. These results are replicated using a more rigid method in experiment 2, in which individual adjustment of the prime contrast was performed to ensure maximal stimulus strength even under full suppression (Shanks 2016; Stein et al. 2020). This difference between numerical processing after implicitly presented ordered vs. non-ordered sequences suggests that order is inattentively processed and may even affect the anticipation of subsequent items.

Only few studies have shown that the processing of non-symbolic quantities can escape CFS and lead to a numerical priming effect and only in small quantities (i.e., subitizing range quantities 1–3; e.g., (Bahrami et al. 2010). For example; Bahrami et al. (2010) showed an identical (but unexpected) linear priming function for non-symbolic invisible primes, symbolic invisible primes, and visible non-symbolic primes. Nonetheless, Hesselmann and Knops (2014) and Hesselmann et al. (2015) challenged the interpretation proposed by Bahrami et al. (2010). After replicating these findings and by using additional analysis, Hesselmann and Knops (2014) and Hesselmann et al. (2015) showed that these priming effects reflect evidence for identity priming (e.g., prime = 3, target = 3) but do not provide conclusive evidence for distance-dependent numerical priming under CFS. Contrary to these conclusions, the results of the current study suggest order-depended numerical priming under CFS. A possible reason for this inconsistency in results is that ratio/distance prime effect was examined, but an underlying ordinality effect affected the results. As we manipulated both ratio and order, we were able to examine whether the numerical or ordinal features of numerosity is implicitly processed under CFS. As our results indicate, it is order and not ratio of the sequences that implicitly facilitates a faster response.

It was previously shown that order and numerosity may be distinct on both the behavioral and the neural level (Sury and Rubinsten 2019). Other studies have found the ability to process ordinality in primates (e.g., Cantlon and Brannon 2006) and in other animal species phylogenetically distant from humans, such as rats (Suzuki and Kobayashi 2000) and parrots (Pepperberg 2006). Infants’ ability to detect ordinality was found for size-based sequences (Cassia et al. 2012), arbitrary sequences of different items (e.g., Lewkowicz and Berent 2009), and quantities (e.g., Brannon 2002; vanMarle 2013). Moreover, the spatial component of ordinal representation (months and letters) is automatically activated (Gevers et al. 2003). Finally, the understanding of order has been found to explain unique variance in children’s arithmetic abilities (e.g., Vogel et al. 2017; Sommerauer et al. 2020). These findings suggest the existence of a distinct processing of order and numerosity. Hence, by manipulating both ratio and order of the primes, our work lays the foundation for a fresh perspective on the role of ordinality in non-symbolic numerical processing.

The relationships between quantities (i.e., order) are implicitly processed even when irrelevant for the task at hand. Hence, order acts as a fundamental numerical characteristic. Furthermore, the process of generating a response (in this case, numerical enumeration) is affected by the implicitly processed order. Referring once again to previous findings that have suggested that order processing appears early in human life (Brannon 2002; Cassia et al. 2012; Cooper and Sophian 1984; de Hevia 2021; de Hevia et al. 2017; de Hevia and Spelke 2010; Feigenson et al. 2002; Picozzi et al. 2010; Suanda et al. 2008), our results suggest that order is a basic feature of the environment to which humans seem wired to attend to by implicitly extracting approximate order.

Schacter (1987) and Reber (1989) claimed that implicit processes are phylogenetically primitive systems. Reber (1989) classified implicit mentation into two classes, *primitive* and *sophisticated*, and defined primitive processes as basic functions that are carried out automatically and are fundamental for a species’ survival. If we follow this line of reason, then current findings suggest the robust impact of implicitly perceived order and provide essential information about the structure of the world around us, information that is essential for a variety of cognitive skills and representations, specifically numerical representation.

The work by Moors et al. (2017) may suggest an alternative explanation for the “order effect” found in the current study. Moors and colleagues (2017) reviewed several recent findings on the neural basis of interocular suppression and suggested that representation of CFS stimuli is not integrated and hence, processing of semantic stimulus features during CFS is not possible. This suggest the possibility that prime sequence was not processed as an integrate and was limited to their basic, elemental features.

Contrary to our research hypothesis, in both experiments the results did not indicate that the ratios of the prime sequences are implicitly processed under interocular suppression (CFS). Thus, order and not cardinality was implicitly processed in both experiments. This “lack” of a ratio effect may be related to the difference in the “distance priming effect” in relation to the notation of the prime. As mentioned earlier, evidence is inconclusive to date for a numerical distance effect under interocular suppression (Bahrami et al. 2010) vs. (Hesselmann et al. 2015; Hesselmann and Knops 2014).

On the other hand, several studies have shown that under visible priming (i.e., unmasked prime presented briefly, so that it is visible but participants have no time to react to it), a distance-dependent priming effect is observed. The distance-dependent priming effect is characterized by a decrease in participant RT (to a target number) as the distance between the prime and the target number decreases (Dehaene et al. 1998; Koechlin et al. 1999). Similar to the distance effect (Krajcsi et al. 2016), “distance-dependent priming effects” are commonly interpreted as reflecting the nature of the “mental number line,” with small numbers represented at one end of the line and larger numbers at the other (Dehaene 1992; Koechlin et al. 1999; Reynvoet et al. 2002).

Roggeman and colleagues (2007) suggested, however, that the priming effect depends on the notation of the prime. These authors compared the “distance-dependent priming effects” of symbolic and non-symbolic numbers in a naming task (participants had to name a symbolic or non-symbolic quantity of 1–5, preceded by a prime numeral, and the distance between the prime and target was manipulated). The results of this study showed that while V-shaped priming (centered on zero distance between target and prime) was found for symbolic digit primes, a step-like priming function resulted from trials with non-symbolic dot primes (a quantity primes all target values that are smaller than or equal to the prime value). Roggeman et al. (2007) suggested that this difference reflects a difference in the underlying representations of symbolic and non-symbolic numerals. Specifically, the pattern of the non-symbolic priming effect is a result of a summation coding scheme in which a non-symbolic number activates a segment in the number line that includes the complete range of quantities up to the target numeral. The symbolic priming effect is a result of a place coding scheme in which a symbolic number activates a specific position on the number line that also activates the numbers close to the target with decreasing strength.

The hypothesis regarding a summation coding scheme of non-symbolic numerals (Roggeman et al. 2007) might act as a possible interpretation of the fact that in the current study, order but not ratios produced a significant effect; this difference between ratio and order might reflect the underlying representation of non-symbolic quantities. To know or enumerate a single quantity requires using the knowledge of how this quantity evolves or of its composition. Hence, estimating a single quantity reflects an understanding of the relationships between the quantities that construct it. In other words, activating a complete range of quantities up to the target is comparable to activating the relevant ordinal sequence up to the target. Hence, the effect of order might reflect the summation coding scheme of non-symbolic numerals. In turn, this might explain the insignificant ratio effect found in the current study.

This interpretation may also apply to the results of Bahrami et al. (2010). Indeed, their findings indicated that implicitly presented non-symbolic primes induce a priming effect that depends on the numerical distance between prime and target. However, the specific pattern of the priming effect was not concordant with previous numerical priming studies. Specifically, RTs were slower (relative to no-prime baseline) when primes were larger than the target (e.g., prime = 3, target = 1) and faster when primes were smaller than the target (e.g., prime = 1, target = 3). A smaller “facilitation” was found when primes and targets were identical (e.g., prime = 3, target = 3).

Considering the summation coding hypothesis, one could argue that the “facilitation” effect found for primes smaller that target trials (Bahrami et al. 2010) results from order-based processes. That is, primes smaller than target trials reflect an increasing order compatible with the numerical sequence. An alternative explanation comes from the findings of studies with human infants that the sensitivity to numerical ordinality is dependent on the ratio of the sequence (e.g., Cassia et al. 2012; Suanda et al. 2008). Hence, it is possible to assume that without a “noticeable difference” between quantities, there will be no effect of ordinality. In other words, to process the relationships within a sequence, one must perceive there is a difference between items in a sequence.

A second alternative explanation for the lack of a ratio effect is related to the number of dots presented in each of the prime sequence stimuli (i.e., the quantities); in a small set of the prime sequences, the quantities 2–3 appeared. These quantities are within the subitizing range. The question of how subitizing operates is debated (Krajcsi et al. 2013). Accordingly, some researchers suggest that since sets with less than five objects can be enumerated fast (subitizing), subitizing is not an ANS directed process and is more likely to be based on pattern detection. Therefore, to measure the approximate number system based dot estimation, the 1–4 range should be avoided (Krajcsi et al. 2018). Thus, a possible explanation for the absence of a ratio effect in our study is the involvement of both subitizing and ANS quantities in the same prime sequences. Yet, it should be highlighted that recent research suggests that subitizing may be consider as processed by the approximate number system. It had been shown that only the relation between symbolic and non-symbolic processing may be stronger within the subitizing range, but both large and small quantities are processed under the approximate number system (Hutchison et al. 2020). Indeed, recent brain imaging studies show a continuum of cortical representation of small (subitizing) and large numerosities, which argues for a single numerosity neural representation mechanism, in line with the single enumeration system of the approximate number system (Cai et al. 2021). The fast and accurate perception that is seen on small numerosities is probably only because more cortical area of the numerosity maps are devoted to smaller numerosities (Harvey and Dumoulin 2017).

Similar to ratio, the results did not indicate that the direction of the prime sequences is implicitly processed under interocular suppression (CFS). Using an explicit order judging task, Rubinsten and colleagues (Rubinsten et al. 2013; Rubinsten and Sury 2011) did find an effect of direction. In these studies, the direction of the sequence affected performance in ordered judgment tasks. Specifically, the effect of order (i.e., a faster and more accurate response to ordinal sequences) was mainly significant in the large to small (descending) direction, compatible with the right to left direction of the Hebrew writing system (participants in these studies were Hebrew-speaking Israelis). The direction of the writing system seems, therefore, to modulate the response to ordinal sequences, and presents a cultural linguistic effect. The insignificant effect of direction implies that symbolic, culturally dependent features such as the direction of the sequence were not implicitly processed. An alternative explanation to the ‘absence’ direction effect in the current study may relate to the language of participants. Specifically, participants in the current study were Hebrew-speaking who read and write letters from right to left but use Arabic numbers from left to right. This dissociation between the reading and writing direction of numbers and letters may result in a sort of flexibility in the directional bias of the mental number line. Specifically, findings of a Spatial Numerical Association of Response Codes Effect [(i.e., SNARC effect which refers to the left to right directional bias of the mental number line (Dehaene et al. 1993)] are inconsistent in Hebrew-speaking participants, with some studies arguing for the absence of the horizontal SNARC (or reversed SNARC) effect (e.g., Fischer et al. 2009, 2010; Shaki et al. 2009; Wood et al. 2008), and others reporting a horizontal, left-to-right SNARC effect under certain experimental conditions (e.g., Zohar-Shai et al. 2017), suggesting cultural factors, such as the direction of reading and writing, influence numerical representation (Göbel et al. 2011; Shaki et al. 2012). Hence, the absence of a direction effect in the current study may be attributed to the language of participants. Further studies may examine whether direction of the sequence is implicitly processed when participants use both numbers and letters in the same direction or even compare processing patterns of different languages. Yet, by manipulating both ratio and order of the primes, using a solid statistical analysis, having a strong statistical power, and thus demonstrating the absence of any ratio effect, our work opens the door to a fresh perspective on the role of ordinality in non-symbolic numerical processing. Future studies should focus on the dissociation of order and ratio in non-symbolic numerical sequences, examining the appropriate method to untangle these complex relationships and consider the role of ordinality as (possibly) a domain general system not exclusive to numbers.

In summary, the implicit priming effect of order and not of quantity or direction found in the current study may reflect both the salience of order in non-symbolic numerals (and not just in symbolic numbers) and additionally have implications regarding the difference between symbolic and non-symbolic numerical representation. As such, order may be accounted for as a possible explanation for the different numerical priming effects found in previous studies.

## 5. Conclusions

Research conducted in recent decades has shown that many perceptual abilities that are highly important for survival (e.g., face recognition, movement perception) are involved in the processing of order. However, the view of order as a common thread between various fields of cognition has been neglected. The current study demonstrates that order is implicitly and inattentively perceived so to affect numerical enumeration even if irrelevant for the task (see De Houwer and Moors 2012). These findings support the notion that implicit order processing is involved in yet one more perceptual ability and call for a deeper investigation into an implicitly based order input analyzer involved in different cognitive functions.

Specifically, in the numerical domain, the current results suggest that estimation of a quantity or even comparison of two quantities requires knowledge about how a number evolves and of its composition. That is, it requires a sense of order within the numerical sequence. Hence, the order of quantities may indeed play a fundamental role in numerical cognition and in how symbolic and non-symbolic representations differ and develop. For non-symbolic numerals, order may be at the heart of subsequently acquired mathematical intelligence.

## Figures and Tables

**Table 1:** Detailed numerical value of the dot patterns in the prime and target stimuli.

Ratio	0.7				0.5			
	Sequence presented as prime			Target	Sequence presented as prime			Target
**Position**	First	Second	Third		First	Second	Third	
	3	4	6	9	2	4	8	16
	4	6	9	13	3	6	12	24
	5	7	10	15	4	8	16	32
	6	9	13	19	5	10	20	40

**Table 2:** Mean RTs and deviations of enumeration for each target that appear in the experiment. Standard deviations are shown on brackets.

Target’s Value	RT	Deviations of Enumeration
9	1319 (430)	0.26 (0.17)
13	1367 (406)	0.37 (0.33)
15	1360 (360)	0.31 (0.17)
16	1371 (407)	0.36 (0.30)
19	1353 (410)	0.34 (0.20)
24	1320 (381)	0.40 (0.31)
32	1295 (381)	0.35 (0.20)
40	1281 (357)	0.34 (0.21)

**Table 3:** Multilevel regression models for testing the effect of order on students’ response time.

		Model 1: All Students		Model 2: Unawareness Subgroup	
		Estimate	S.E.	Estimate	S.E.
Level One	Order	−0.014 ~	0.009	−0.023 *	0.012
	Ratio	−0.059 *	0.029	−0.062 ~	0.036
	Residual Variance	0.232 ***	0.020	0.250 ***	0.029
Level Two	Accuracy	−0.004	0.003	−0.009 ~	0.006
	Mean Response Time ^1^	1.558 ***	0.163	1.810 ***	0.282
	Residual Variance	0.100 ***	0.021	0.126 ***	0.033
Sample N		69		41	
Model Fit	AIC = 13,113; BIC = 13,137		AIC = 904; BIC = 9115		

^1^ Response Time/1000; ~ *p* < .10, * *p* < .05, ** *p* < .01, *** *p* < .001.

**Table 4:** Experiment 2—Mean RTs and deviations of enumeration for each target that appear in the experiment. Standard deviations are shown on brackets.

Target’s Value	RT	Deviations of Enumeration
9	1414 (413)	0.30 (0.23)
13	1479 (454)	0.38 (0.25)
15	1471 (463)	0.33 (0.22)
16	1445 (404)	0.33 (0.21)
19	1409 (420)	0.31 (0.22)
24	1414 (361)	0.31 (0.18)
32	1357 (379)	0.29 (0.17)
40	1335 (387)	0.25 (0.13)

## Data Availability

The data presented in this study are available on request from the corresponding author. The data are not publicly available due to privacy restrictions.

## Appendix A. Description of Non-Symbolic Numerical Stimuli

Stimuli were generated using a custom-written software programmed in the C# (pronounced as c sharp) language above Microsoft. NET 2 Framework™ using Visual Studio 2005 IDE. This software provided control of parameters of the dot patterns. We used a resolution of 1024 × 768 to create the images. Dot location in each stimulus was randomized and consisted of random density and area of dots (see Figure 1 for illustration).

White dots appeared on a black background and were positioned within the bounds of a white circle of a 3° visual angle (VA) (VA calculated using the formula: where *d* is the distance between the subject’s eye and the screen and s is the size of the object on the screen). Each dot size was randomly varied between 0.17–1.5° VA. Each dot’s position was determined by placing it on a randomized arc of an inner circle with a randomized radius using the following formula: *d*(*x*,*y*) = [*r* sin(*α*),*r* cos(*α*)] where *d*(*x*,*y*) is the dot position on *x*,*y* space, *r* is a randomized radius from the center of the invisible circle, and *α* is a randomized arc (0–360°) on a circle created with this radius. The randomization method relies on the system’s random library which provides a pseudo random number generation method. Each dot was smoothed using the advanced anti-aliasing algorithm provided with the Microsoft.Graphics2D code library.

The dots never touched each other and were no closer than 0.1 VA. This was achieved by randomly selecting a dot location (see randomizing dot location) and comparing the distance of this dot to all the others. If the dot was not closer to another dot than a fixed minimum (1-degree VA), the dot was painted. Otherwise, a new position would have been randomly selected. A maximum number of iterations (5000) were determined as a stop criterion. When the criteria were not met, the stimuli were omitted and the program wrote an error message for not being able to create a suitable dot array for this numerosity.

## Appendix B. Detailed Description of ICC Method

The intra-class correlation coefficient measured the ratio between the student-level variance to the total explained variance. Thus, the ICC is the correlation among measurements within the same cluster of questions (ICC(1): (MSB − MSW)/(MSB + (k − 1)*MSW)). This is a common practice in analyzing multi-level data, that is, repeatedly measured participants’ responses (Snijders and Bosker 1999; Bickel 2007). A larger than 0.05 ratio (usually, a rule of thumb) means that a significant portion of the data could potentially be explained by variability between participants beyond the variability across participants’ answers. More specifically, the design effect of the large cluster size (number of repeated questions per student) was significantly higher than the necessary threshold of two (Muthén and Satorra 1995), in addition to the fact that the two-level analyses were the result of the experimental design, namely a by-design two-level structure of data. The ICC was used, to assess the potential of level two (subjects) data in explaining the variance of the outcome variable (i.e., reaction time). Formally, multilevel data mean that the outcome measure varies between subjects and between measurements, that is, the model includes a random intercept as well as random slopes of explanatory variables: y_ij_ = α_j_ + b_j_x + u_j_ + ε_ij_, where i is index of measurements and j is an index of students, α and b are model estimates, which randomly vary across students. The design matrix included subjects times constant plus explanatory variables: **X** = {c_1_, c_2_, …, c_n_, x_1_, x_2_, …, x_n_}, where c stands for intercept and x stands for independent variables.
